# Hospital-Based Same-Day Compared to Overnight-Stay Mastectomy: An American College of Surgeons National Surgical Quality Improvement Program Analysis

**DOI:** 10.31486/toj.21.0103

**Published:** 2022

**Authors:** Udai S. Sibia, John R. Klune, Justin J. Turcotte, Luther H. Holton, Adam I. Riker

**Affiliations:** ^1^ Department of Surgery, Anne Arundel Medical Center at Luminis Health, Annapolis, MD; ^2^ Department of Oncology, Anne Arundel Medical Center at Luminis Health, Annapolis, MD

**Keywords:** *Ambulatory surgical procedures*, *enhanced recovery after surgery*, *length of stay*, *postoperative complications*, *same-day mastectomy*, *treatment outcome*

## Abstract

**Background:** Enhanced Recovery after Surgery for mastectomy has resulted in increased use of outpatient same-day mastectomy (SDM). Whether SDM leads to increased readmissions or reoperations is not well documented. This study examines national data to compare outcomes of SDM to an overnight stay.

**Methods:** We analyzed the American College of Surgeons National Surgical Quality Improvement Program Participant Use Data File from 2016 to 2018 for all mastectomy cases. Cases with a length of stay (LOS) >1 day were excluded. Cases were then categorized into 2 LOS cohorts: SDM vs 1-day LOS.

**Results:** A total of 22,642 cases (80.8% 1-day LOS vs 19.2% SDM) were identified for the final analysis. Patients in the 1-day LOS group were more likely to be older (57.9 vs 54.0 years, *P*<0.01), be female (98.0% vs 79.8%, *P*<0.01), and have greater comorbidity (38.1% vs 30.7% American Society of Anesthesiologists classification 3 or 4, *P*<0.01) compared to the SDM group. Multivariate analysis demonstrated no difference in risk for 30-day wound complications between the SDM and 1-day LOS groups. The risks for 30-day medical complications (1.60 odds ratio [OR], 95% CI 1.06-2.42, *P*=0.02), reoperations (1.46 OR, 95% CI 1.17-1.81, *P*<0.01), and readmissions (1.60 OR, 95% CI 1.25-2.05, *P*<0.01) were higher in the 1-day LOS group. Even after excluding patients undergoing reoperation on the day of surgery, the risk for reoperations (2.3% vs 3.3%, *P*<0.01) remained higher in the 1-day LOS group. Characteristics associated with 1-day LOS were hypertension, steroid use, diabetes, dyspnea, dependent functional status, bilateral procedures, and breast reconstruction.

**Conclusion:** We demonstrate that SDM is a safe procedure, with no increase in risk for 30-day postoperative complications. Appropriate patients should be offered SDM.

## INTRODUCTION

Breast cancer is the most common malignancy among women in the United States, with an estimated 276,480 new cases diagnosed in 2020.^1^ Nearly 33.8% of these patients undergo a mastectomy.^2^ The safety of outpatient mastectomy has been well documented.^3-9^ The majority of patients undergoing outpatient mastectomy, with or without breast reconstruction, will stay overnight for up to a 23-hour observation period. Enhanced Recovery after Surgery (ERAS) for mastectomy has resulted in increased use of outpatient same-day mastectomy (SDM). SDM protocols have been described^10,11^ but have not been widely adopted across health care systems in the United States.

The coronavirus disease 2019 pandemic has driven health care systems toward delivering more efficient, less costly, and safer patient care. The pandemic forced many institutions to re-examine their approach and criteria for patients who truly require an overnight hospital stay, and this effort increased interest in developing and implementing SDM protocols that minimize the need for an overnight hospital stay.^12-16^ However, whether the safety of SDM demonstrated at single integrated health care centers is generalizable to other institutions at the national level remains unclear. The primary purpose of this study was to examine 30-day outcomes of SDM using a large national database. The secondary outcome was to identify patient and operative factors associated with 1-day length of stay (LOS).

## METHODS

### Patients

We obtained institutional review board approval for this retrospective analysis utilizing the American College of Surgeons National Surgical Quality Improvement Program (ACS NSQIP) Participant Use Data File from 2016 to 2018. Mastectomy cases were selected using Current Procedural Terminology codes 19303 and 19304 (simple, subcutaneous). We included elective procedures and only those with clean wounds (wound class I). Emergency cases and patients admitted with sepsis were excluded. We excluded cases with an LOS >1 day as these patients were likely to either have comorbidities that rendered them ineligible for SDM or may have already experienced a complication. Therefore, cases with an LOS >1 day were excluded to avoid bias to our data. The total number of eligible cases for this analysis was 22,642. Cases were categorized into 2 groups: SDM vs 1-day LOS. SDM was defined as patients discharged on the day of surgery, while 1-day LOS was defined as patients who stayed overnight and were discharged on postoperative day 1.

### Outcomes

The primary outcomes were 30-day total wound and medical complications, readmissions, and reoperations. Wound complications included superficial incisional surgical site infection (SSI), deep incisional SSI, organ space SSI, and wound disruption. Medical complications were defined as pneumonia, pulmonary embolism, urinary tract infection, cerebrovascular accident, myocardial infarction, bleeding transfusions, deep vein thrombosis/thrombophlebitis, sepsis, septic shock, reintubation, failure to wean off ventilator >48 hours, renal insufficiency, renal failure, and cardiac arrest requiring cardiopulmonary resuscitation. Secondary outcomes were patient and operative factors associated with 1-day LOS. The ACS NSQIP database does not collect hospital costs as a variable. We were also unable to collect our institutional cost data to provide any meaningful cost analysis of SDM vs 1-day LOS.

### Statistical Analysis

We first analyzed the data by examining for any outliers and overall general trends. Next, descriptive statistics were used to examine the distribution of each outcome of interest. The Pearson chi-squared and two-sided Fisher exact tests analyzed differences in categorical variables between groups. Categorical variables included sex, race, comorbidity (diabetes mellitus, smoking history, dyspnea, chronic obstructive pulmonary disease, congestive heart failure, hypertension, dialysis, disseminated cancer, chronic steroid use, >10% weight loss in <6 months, and bleeding disorders), functional status, American Society of Anesthesiologists (ASA) classification, and 30-day postoperative complications. We used *t* test to analyze differences in continuous variables (ie, age). Multivariate analysis controlled for differences in comorbidity between groups and identified patient and procedure characteristics associated with 1-day LOS. All analyses were performed using SPSS Statistics, version 25 (IBM). A *P* value ≤0.05 was treated as statistically significant.

## RESULTS

A total of 22,642 cases (80.8% 1-day LOS vs 19.2% SDM) were identified for the final analysis. Patients in the 1-day LOS group were more likely to be older (57.9 vs 54.0 years, *P*<0.01), be female (98.0% vs 79.8%, *P*<0.01), and have greater comorbidity (38.1% vs 30.7% ASA classification 3 or 4, *P*<0.01) compared to the SDM group (Table 1). The 1-day LOS group was more likely to undergo simple mastectomy, bilateral procedures, or have immediate breast reconstruction (Table 2).

**Table 1.** Patient Demographics

Variable	Same-Day Mastectomy Group, n=4,357	1-Day Length of Stay Group, n=18,285	*P* Value
Age, years, mean	54.0	57.9	<0.01
Sex			<0.01
Male	880 (20.2)	357 (2.0)	
Female	3,477 (79.8)	17,928 (98.0)	
Race			<0.01
White	2,391 (54.9)	13,621 (74.5)	
Black or African American	313 (7.2)	1,674 (9.2)	
Asian	335 (7.7)	921 (5.0)	
Unknown/Not reported	1,263 (29.0)	1,941 (10.6)	
Native Hawaiian or Pacific Islander	45 (1.0)	66 (0.4)	
American Indian or Alaska Native	10 (0.2)	62 (0.3)	
Comorbidities			
Diabetes mellitus	471 (10.8)	2,172 (11.9)	0.04
Current smoker within 1 year	550 (12.6)	2,015 (11.0)	<0.01
Dyspnea with moderate exercise or at rest	150 (3.4)	769 (4.2)	0.02
Chronic obstructive pulmonary disease	95 (2.2)	464 (2.5)	0.17
Congestive heart failure within 30 days	11 (0.3)	35 (0.2)	0.42
Hypertension requiring medication	1,469 (33.7)	6,924 (37.9)	<0.01
Currently on dialysis	11 (0.3)	28 (0.2)	0.16
Disseminated cancer	60 (1.4)	269 (1.5)	0.64
Steroid use for chronic condition	76 (1.7)	417 (2.3)	0.03
>10% weight loss in <6 months	15 (0.3)	67 (0.4)	0.83
Bleeding disorders	43 (1.0)	219 (1.2)	0.24
Functional status^a^			0.02
Independent	4,306 (99.4)	18,043 (99.1)	
Partially or totally dependent	24 (0.6)	165 (0.9)	
American Society of Anesthesiologists classification^b^			<0.01
1	643 (14.8)	868 (4.8)	
2	2,369 (54.4)	10,426 (57.1)	
3	1,279 (29.4)	6,737 (36.9)	
4	59 (1.4)	227 (1.2)	

Note: Data are reported as n (%) unless otherwise indicated.

^a^n=4,330 for the same-day group and n=18,208 for the 1-day length of stay group.

^b^n=4,350 for the same-day group and n=18,258 for the 1-day length of stay group.

**Table 2.** Procedure Details

Variable	Same-Day Mastectomy Group, n=4,357	1-Day Length of Stay Group, n=18,285	*P* Value
Mastectomy			<0.01
Simple	3,546 (81.4)	17,737 (97.0)	
Subcutaneous	811 (18.6)	548 (3.0)	
Laterality			<0.01
Unilateral	3,640 (83.5)	13,680 (74.8)	
Bilateral	717 (16.5)	4,605 (25.2)	
Breast reconstruction			<0.01
Immediate	214 (4.9)	1,493 (8.2)	
Delayed	268 (6.2)	5,990 (32.8)	

Note: Data are reported as n (%).

Univariate analysis demonstrated an increased risk of 30-day total wound and medical complications, readmissions, and reoperations in the 1-day LOS group compared to the SDM group (Table 3). The increased risk in wound complications was attributable to superficial incisional and organ space SSI. Multivariate analysis, controlling for differences in comorbidity between the 2 groups, revealed no difference in risk for superficial incisional SSI, organ space SSI, or total wound complications (Table 4). The risk for total medical complications, reoperations, and readmissions remained higher for patients in the 1-day LOS group.

**Table 3.** Univariate Analysis of Postoperative Complications

Complication	Same-Day Mastectomy Group, n=4,357	1-Day Length of Stay Group, n=18,285	*P* Value
Any wound complication within 30 days	113 (2.6)	637 (3.5)	<0.01
Superficial incisional surgical site infection	62 (1.4)	348 (1.9)	0.03
Organ space surgical site infection	22 (0.5)	160 (0.9)	0.01
Deep incisional surgical site infection	17 (0.4)	83 (0.5)	0.57
Wound disruption	13 (0.3)	69 (0.4)	0.44
Any medical complication within 30 days	28 (0.6)	202 (1.1)	<0.01
Urinary tract infection	10 (0.2)	38 (0.2)	0.78
Sepsis	7 (0.2)	65 (0.4)	0.04
Myocardial infarction	3 (0.1)	8 (0.0)	0.50
Bleeding transfusions	5 (0.1)	17 (0.1)	0.68
Deep vein thrombosis/thrombophlebitis	3 (0.1)	23 (0.1)	0.32
Septic shock	3 (0.1)	6 (0.0)	0.24
Pneumonia	2 (0.0)	16 (0.1)	0.38
Pulmonary embolism	1 (0.0)	20 (0.1)	0.09
Cerebrovascular accident/stroke with neurologic deficit	0 (0.0)	11 (0.1)	0.11
Reintubation	1 (0.0)	7 (0.0)	0.53
Failure to wean off ventilator >48 h	2 (0.0)	4 (0.0)	0.33
Renal insufficiency	2 (0.0)	2 (0.0)	0.17
Renal failure	0 (0.0)	3 (0.0)	0.53
Cardiac arrest requiring cardiopulmonary resuscitation	1 (0.0)	2 (0.0)	0.47
30-day return to the operating room	110 (2.5)	664 (3.6)	<0.01
30-day readmission	81 (1.9)	555 (3.0)	<0.01

Note: Data are reported as n (%).

**Table 4.** Multivariate Analysis Demonstrating the Odds for Postoperative Complications in the 1-Day Length of Stay Group

		95% CI		
Variable	Exp(B)	Low	High	*P* Value
Any wound complication within 30 days	1.21	0.98	1.50	0.07
Superficial incisional surgical site infection	1.24	0.93	1.65	0.14
Organ space surgical site infection	1.53	0.96	2.42	0.07
Any medical complication within 30 days	1.60	1.06	2.42	0.02
30-day return to the operating room	1.46	1.17	1.81	<0.01
30-day readmission	1.60	1.25	2.05	<0.01

Exp(B), exponentiation of the B coefficient.

To avoid risk of bias by including patients with complications in the 1-day LOS group, we performed a subset analysis excluding patients with a day-of-surgery return to the operating room. Even after excluding these patients, the risk of reoperations remained higher in the 1-day LOS group (2.3% vs 3.3%, *P*<0.01) (data not shown).

Smoking, diabetes mellitus, dyspnea with moderate exercise or at rest, and hypertension requiring medication were identified as risk factors for wound complications (Table 5). Dyspnea with moderate exercise or at rest and hypertension requiring medication were associated with increased risk for medical complications. Smoking also increased the risk for 30-day reoperations and readmissions. Other risk factors for readmission included partially or totally dependent functional status, dyspnea with moderate exercise or at rest, and hypertension requiring medication.

**Table 5.** Multivariate Analysis Identifying Risk Factors for Postoperative Complications

		95% CI		
Variable	Exp(B)	Low	High	*P* Value
Risk factors for wound complications within 30 days				
Smoking	1.88	1.56	2.28	<0.01
Diabetes mellitus	1.51	1.22	1.85	<0.01
Dyspnea with moderate exercise or at rest	1.36	1.00	1.86	0.05
Hypertension requiring medication	1.35	1.14	1.60	<0.01
Risk factors for medical complications within 30 days				
Dyspnea with moderate exercise or at rest	2.21	1.41	3.47	<0.01
Hypertension requiring medication	1.42	1.05	1.92	0.02
Risk factor for 30-day return to the operating room				
Smoking	1.33	1.08	1.63	0.01
Risk factors for 30-day readmission				
Partially or totally dependent functional status	2.62	1.57	4.37	<0.01
Dyspnea with moderate exercise or at rest	1.98	1.47	2.67	<0.01
Smoking	1.36	1.08	1.70	<0.01
Hypertension requiring medication	1.28	1.07	1.54	<0.01

Exp(B), exponentiation of the B coefficient.

Patient characteristics associated with 1-day LOS were partially or totally dependent functional status, hypertension requiring medication, steroid use within 30 days, dyspnea with moderate exercise or at rest, and diabetes mellitus (Table 6). Operative characteristics associated with 1-day LOS were bilateral procedures and breast reconstruction, both immediate and delayed.

**Table 6.** Multivariate Analysis Identifying Patient and Operative Characteristics Associated With 1-Day Length of Stay

		95% CI		
Variable	Exp(B)	Low	High	*P* Value
Patient characteristics				
Partially or totally dependent functional status	1.79	1.14	2.79	0.01
Hypertension requiring medication	1.32	1.22	1.44	<0.01
Steroid use within 30 days	1.31	1.01	1.69	0.04
Dyspnea with moderate exercise or at rest	1.27	1.05	1.53	0.01
Diabetes mellitus	1.18	1.04	1.32	<0.01
Operative characteristics				
Delayed breast reconstruction	6.86	6.00	7.85	<0.01
Immediate breast reconstruction	1.88	1.61	2.20	<0.01
Bilateral mastectomy	1.21	1.09	1.33	<0.01

Exp(B), exponentiation of the B coefficient.

## DISCUSSION

The Kaiser Permanente Northern California health care system was among the first to implement an SDM protocol across its 21 medical centers.^10^ Kaiser Permanente successfully increased the utilization of SDM from 16% to 75% during the course of a year, without any corresponding increase in emergency department visits, readmissions, or reoperations.^10^ Another initiative across 13 hospitals in Canada demonstrated an increase in SDM utilization from 1.7% in 2011 to 47.8% in 2018, with no corresponding increase in either readmissions or reoperations.^11^ Patient-reported experience measures surveys showed an overall 90% level of satisfaction with SDM planning and ability to self-care and recover at home.^11^

Several other single institutions have reviewed the safety of their SDM protocols and have reported similar advantages of shorter LOS and lower hospital costs.^12-16^ These smaller studies have been somewhat limited in their findings as single-center experiences. Thus, the question is if these favorable outcomes with SDM can be widely applied and implemented at the national level. This study analyzed a large national database and reaffirmed that SDM is not associated with a higher likelihood of an adverse postoperative event or outcome. In fact, the risk of total medical complications, reoperations, and readmissions was lower for patients in the SDM group compared to those who stayed overnight. One possible explanation could be that SDM patients who require reoperation on the day of surgery are more likely to stay overnight for additional monitoring and would therefore affect the data. However, our analysis controlled for patients undergoing a reoperation on the day of surgery and still found an elevated risk for reoperations in the 1-day LOS group.

At breast centers considering SDM protocols, the use of a multimodal approach to pain management, beginning in the preoperative setting and extending through same-day discharge to home, should be evaluated. A pain management regimen may include a variety of medications, including preoperative gabapentin, acetaminophen, nonsteroidal anti-inflammatory drugs (NSAIDs), and scopolamine. Intraoperative regional nerve blocks can be successfully achieved with long-acting anesthetics, such as liposomal bupivacaine via a multilevel midaxillary intercostal nerve block.^12^ A multimodal approach to pain management has been shown to result in significantly lower pain scores in the recovery room, reduce perioperative opioid consumption, decrease postoperative nausea and vomiting, and shorten LOS.^17-21^

Several patient characteristics are associated with 1-day LOS. Partially or totally dependent patients are likely to remain in the hospital longer because of their increased need for support to complete activities of daily living.^22^ Cognitive impairment prior to surgery and decline in activities of daily living prior to surgery are both independent risk factors associated with 1-year functional decline after breast cancer surgery.^22^ Preoperative shoulder range of motion exercises reduce postoperative pain and expedite recovery after mastectomy.^23^ Diabetes has also been shown to increase the risk of postoperative complications and lengthen LOS for patients undergoing breast reconstruction.^24^

Operative characteristics correlating with 1-day LOS were bilateral procedures and breast reconstruction. We believe that the main reason for an overnight hospital stay is the concern for patient safety. Studies have shown that admitting breast reconstruction patients overnight does not prevent short-term complications, and early discharge has been shown to be quite safe in this population.^25-27^ Neoadjuvant chemotherapy, immediate breast reconstruction, or bilateral mastectomy does not increase the risk for hospital readmissions.^28^

The above findings are consistent with a 2021 study that identified increased age, ASA class 3 or 4, bilateral operation, immediate reconstruction, estimated blood loss >100 mL, perioperative NSAIDs, and perioperative gabapentin to be associated with a lower likelihood for SDM.^28^ High-volume breast surgeons who use perioperative intravenous acetaminophen and opioids in ERAS protocols increase the likelihood of SDM.^28^

Our institutional patient selection criteria for SDM include unilateral or bilateral mastectomy, with or without sentinel lymph node biopsy; simple or complete mastectomy; and modified radical mastectomy, with or without immediate breast reconstruction. Patients excluded from our SDM protocol include those undergoing free-flap breast reconstruction and patients with significant medical comorbidities and/or inadequate family support systems. A case-by-case decision is made for patients on anticoagulation, on neoadjuvant chemotherapy, or with extensive disease burden in the axilla.

Our study has limitations. The ACS NSQIP database is only a cross-section of all mastectomy cases performed nationally each year. The 2018 dataset contained 1,020,511 cases submitted from 722 participating sites.^29^ As such, we believe that the dataset is a reliable source to investigate outcomes after SDM at the national level. As with any retrospective database study, we have no means of identifying the intention-to-treat grouping for cases. Consequently, confounding factors may exist that we were unable to control for in this study. Additionally, selection bias is likely regarding the patients eligible for SDM, as SDM is still in an early adoption phase, and only optimal candidates are probably being selected for SDM. Finally, the ACS NSQIP does not collect or report hospital costs. We were also unable to collect our institutional cost data to provide any meaningful cost analysis of SDM vs 1-day LOS.

## CONCLUSION

This study demonstrates that SDM is a safe procedure, with no increase in risk for adverse 30-day postoperative complications. The risk of total wound complications was similar between the SDM and 1-day LOS groups. The risk for 30-day medical complications, readmissions, and reoperations was lower in the SDM group compared to the 1-day LOS group. Bilateral procedures, inclusive of immediate breast reconstruction, can be safely performed as an SDM procedure.

## Notes

*This article meets the Accreditation Council for Graduate Medical Education and the American Board of Medical Specialties Maintenance of Certification competencies for Patient Care, Medical Knowledge, and Practice-Based Learning and Improvement.*
